# Statistical study on the environmental effects on the natural variation of nutritional components in rice varieties

**DOI:** 10.1002/fsn3.839

**Published:** 2018-10-30

**Authors:** Seon‐Woo Oh, Soyoung Lee, Sooyun Park, Sanggu Lee, Seongkon Lee, Hyunsuk Cho, Youngsoo Chung, Soonki Park

**Affiliations:** ^1^ National Institute of Agricultural Science Rural Development Administration Jeonju‐si Korea; ^2^ Department of Molecular Genetic Engineering Dong‐A University Pusan Korea; ^3^ School of Applied Biosciences Kyungpook National University Daegu Korea

**Keywords:** assessment, environmental effect, GMO, natural variation, nutrients

## Abstract

This study was investigated to compare the natural variation of nutrients in rice variety by different environmental factors. Fifteen kinds of rices were used, which were cultivated in two locations for 2 years. All data were analyzed by the various statistical tools to identify the nutritional variations of nutrients. The results of variable importance in the prediction analysis were found to be consistent with the % variability. The nutrient compositions most affected by variety were fatty acids, and next were vitamins, proximate nutrients, minerals, and amino acids in order. The nutrient compositions most affected by location were proximate, followed by minerals, vitamins, fatty acids, and amino acids. For cultivation year, vitamins were most affected and then minerals, fatty acids, proximate nutrients, and amino acids in order. These findings could explain that each kind of nutrients can be naturally varied by different environmental factors.

## 1 INTRODUCTION

Informative big data of plants nutritional compositions are very important for the nutritional safety assessment of new biotechnology organisms. Also, these are useful to the food industry and food‐based dietary guidelines for different sources of food nutrients. Furthermore, the nutritional compositions of commercial plants can play an important role by providing information on natural chemical components as well as the presence and amounts of interacting components. Specifically, for the safety assessment of new biotechnology organisms such as genetically modified organisms (GMOs), the level of nutritional compositions of commercial crops is considered as references for the identification of significant compositional changes in GMOs (EFSA, 2010). Because of the natural compositional change in commercial crops, it is necessary to study whether the component change due to other biotechnology belongs to this natural variation category or not. Natural variation of the components in crops has been reported in several articles (Nicole & Daniel, 2015; Thomas, Richard, Howard, Eugenia, & Karl, 2012). Statistical methods for compositional assessment can be used to identify natural variability differences observed from plant varieties or other variables. The natural variation of plants nutritional composition can be influenced by a number of environmental factors such as cultivation location and plant varieties, many of which can be barely controlled and induced natural variations. Also the micronutrients content and bioactive substances can be mainly affected by the seasonal variations in plant foods (Collomb et al., 2008). For this reason, the tolerant or confidence interval range of nutritional compositions by statistical tools can be used to improve credibility from obtained experimental data. Tolerance intervals are the most appropriate for evaluating whether or not a transgenic variety is within the normal range for commercial varieties of the same crop. Until now, in order to study the environmental effects of crop components, many researches have been conducted using suitable statistical models. As it was deemed reasonable to interpret integrated statistical models for natural variations of the components by environment, this study was intended to produce comprehensive results by various statistical techniques. All data were suggested by the mean value and standard deviation with the tolerance interval. The natural variation of rice nutrition by using various statistical methods was also investigated. The effects of genotype and environmental variables on the compositional variations were identified through statistics. The environmental factors used were cultivation location, kinds of cultivar, and cultivation year. Rice grain samples (brown rice) were analyzed for their key nutritional components as recommended by OECD consensus document (OECD, 2004). All analyzed data were loaded to the database of web service, “DB for nutritional components of plant foods,” to be used to the GMO safety. All data were compared statistically by using mean and standard deviation.

This study provided information on the natural variation in rice nutrient composition according to various environmental factors, and the results may offer reasonable and scientific assessment in nutritional profiles for future genetic modified of rice or other plants.

## 2 MATERIALS AND METHODS

### 2.1 Rice samples

Fifteen kinds of rice varieties were cultivated at two different geographical locations namely Cheonan and Jeonju in South Korea in 2015 and 2016. Rice varieties were all commercially available with similar genetic background, the “Japonica” type. Varieties used in this study were Keumo (KO), Nakdong (ND), Nampyeong (NP), Dongan (DA), Samkwang (SK), Seolhyangchal (SHC), Sobi (SB), Anmi (AM), Yeongan (YA), Odae (OD), Unkwang (UK), Chilbo (CB), Hiami (HA), Hwaseong (HS), and Hwayoung (HY). These rice varieties were all planted in May and harvested in October in each location. The environmental conditions at these locations are presented in Table 1. Rice varieties were planted at each field sites with a strip‐plot design in five replicates per site. Rice plants were all pretreated for analysis based on the previous research conducted (Oh, Park, Yeo, Park, & Kim, 2015). Whole grain (rough rice) samples were collected from each block and dried at a final moisture concentration of 9%–11% for compositional analysis based on the previous study conducted. The rough rice varieties were manually dehulled using a hulling machine (TR 13) to produce brown rice grain and then finely ground using a planetary mono mill (Pulverisette 6; Fritsch, Germany). The powdered grain of each sample was transferred immediately to −80°C until analysis.

**Table 1:** Environmental conditions of Cheonan and Jeonju in 2015 and 2016

Location	Soil pH^a^	Month	Temp (°C)^b^			Rainfall (mm)^c^	Year
			Min.	Max.	Average		
Cheonan	6.3	5	4.6	31.5	18.4	27.5	2015
		6	12.4	34.2	22.6	86	
		7	13.9	33.2	24.9	136.8	
		8	17.2	36.3	25.3	64.2	
		9	10.6	30.4	20.5	29	
		10	−1.5	25.6	14.5	69	
	6.5	5	5.6	30.4	18.2	107.2	2016
		6	11.7	31.1	22.3	36.2	
		7	18.2	33.3	25	364.3	
		8	14.9	35.1	26	82	
		9	9.9	30.4	21	55	
		10	−2.3	27.5	14.5	95.9	
Jeonju	6.7	5	4.9	33.2	19.2	40.6	2015
		6	12.7	33.6	22.7	124.7	
		7	17	34.2	25.1	121.9	
		8	19.3	35.3	25.9	49	
		9	13.7	31.7	21.6	36.7	
		10	2.1	26.1	16.1	98.1	
	6.7	5	9.6	30.1	19.2	84.3	2016
		6	15.7	31.3	22.8	95.8	
		7	20.4	34.5	26.5	251.8	
		8	16.2	36.2	27.4	35.8	
		9	13.9	32.5	22.6	145.4	
		10	3.3	29.8	16.5	152.3	

a pH value before planting.

b Mean value of daily temperature.

c Total volume of rainfall during 1 month.

### 2.2 Analysis of rice grains nutritional compositions

Rice grains were analyzed for the proximate components (moisture, crude protein, crude fat, ash, crude fiber, soluble dietary fiber [SDF], insoluble dietary fiber [IDF], and carbohydrates), and micro nutrients such as amino acids, minerals, fatty acids, and vitamins. Moisture was measured by gravimetric measurement using hot‐air oven at 105°C. Crude fat was analyzed using Soxhlet extraction method (AOAC, 2000a) while crude protein amount was calculated from total nitrogen content using Kjeldahl method (AOAC, 2005a). Ash content was determined by incinerating the sample in a furnace at 600°C for 22 hr to constant weight (AOAC, 2005b). Carbohydrates were calculated by 100% minus sum of % protein, % lipid, and % ash. Crude fiber content was determined according to AOAC method 962.09 (AOAC, 2005c). Insoluble (IDF) and soluble dietary fiber (SDF) contents were determined by enzymatic‐gravimetric methods using amylase, protease, and amyloglucosidase according to the AOAC method 991.43 (Lee, Prosky, & Devries, 1992). Amino acids were analyzed directly after protein hydrolysis using an automatic amino acid analyzer with hydrochloric acid (AOAC, 2005d). The sulfur‐containing amino acids (cysteine and methionine) were oxidized using performic acid before hydrolysis with 6N hydrochloric acid. The contents of individual amino acids were expressed by % of total protein. Minerals such as copper, iron, zinc, manganese, calcium, sulfur, magnesium, potassium, phosphorus, and sodium were determined using inductively coupled plasma optical emission spectrometry (Inegra XL; GBC Co., Melbourne, Australia) according to the AOAC method 999.11 (AOAC, 2000b). Individual fatty acids were determined according to the AOCS method Ce 1‐62 (Gunstone, 1995) using a Shimadzu GC‐2010 gas chromatograph (Shimadzu, Kyoto, Japan) and expressed by % of total fatty acids. Vitamin B_1_ was assessed according to the method of Sims and Shoemaker with a slight modification (Sims & Shoemaker, 1993). Vitamin B_1_ was extracted from the rice samples (0.1 g) by adding 1 ml 0.1 N hydrochloric acid, vortex mixing for 20 s, and placing in a water bath at 80°C for 30 min. Sep‐Pak C18 cartridges (Waters, Milford, MA) were used for elution of reactive sample solution. Elution was performed using 2 ml of 60%/40% methanol/5 mM ammonium acetate (pH 5.0) mixture and applied to a HPLC analysis. Vitamin B_2_ was analyzed by HPLC fluorometric detection according to the method of Esteve, Farre, Frígola, and Garcıa‐Cantabella (2001) while Vit B_3_ was determined by using a GC TOF MS (Kim et al., 2011). Vitamin B_7_ was determined according to the method of the Korean Food Code (MFDS, 2017) and Vitamin E (α‐tocopherol) using an Agilent 7890A gas chromatograph (Agilent, Atlanta, GA, USA) (Kurilich & Juvik, 1999).

### 2.3 Statistical analysis

The statistical analysis used was SAS 9.2 software package (SAS Institute, Cary, NC, USA) for the significant differences among variables and tolerance interval (TI) of composition data. For each nutrient component of rice varieties, the means and ranges were calculated with five replications per varieties. TIs were suggested for every component to describe the statistical natural ranges in commercial rice. The 99% TI with 95% confidence intervals for each component was assessed using proc capability in the SAS 9.2 software (Lai, Yen, & Chen, 2012). This criterion of confidence level can cover the distribution to fall the interval for the γ‐content of tolerant interval. Negative limits are set to zero. The interaction effect of the variety, location, and year which can influence nutrition variation was also calculated by using the lme4 package of R statistics (version 3.4.3, 2017). With this R package, the linear mixed model was developed for the random effect of data (intercept) and described the distribution of three vector‐valued random variables for variety, location, and year. To complement this statistical model and to estimate the importance of each nutrient variable with metabolite data in rice cultivars, the statistics of the variable importance in the prediction (VIP) value for variables from the PLS‐DA were performed. VIP value was illustrated in a descending order of importance in the nutrient compositions. VIP score is the weighted sum of squares of the PLS loadings. The value of explained Y variance in each dimension influences the weights of sum of PLS regression coefficients (Bijlsma et al., 2006; Xia, Psychogios, Young, & Wishart, 2009). In order to visualize the effect of genetic and environmental backgrounds on nutritional variation, the data were subjected to a principal component analysis (PCA). PCA was performed based on analysis of means using BioPAT‐SIMCA (ver. 13; Umetrics, Umeå, Sweden) to evaluate differences visually among groups of multivariate data. The results of PCA were shown with score plots and loading plot to visualize the contrast between different samples. All data were automatically scaled using unit variance before PCA application. Finally, the two‐way ANOVA by SAS package (9.2; SAS Institute) was used to verify the PCA results of components for more statistical or scientific analysis.

## 3 RESULTS

### 3.1 Nutritional compositions of 15 rice varieties

To investigate the natural variations in the nutritional compositions of 15 commercial rice varieties, the rice varieties were cultivated in the test farms in Cheonan and Jeonju. The nutritional content of each rice variety was analyzed, and the results were presented as mean value and standard deviation (Supporting Information Table S1). The units of each component were adjusted to be the same as those presented in the OECD document (OECD, 2004). The *p*‐value for the significant differences between varieties, two locations, and two cultivation years was identified by ANOVA analysis of SAS package while the tolerance interval ranges were determined using proc capability of the SAS 9.2 software package (Supporting Information Table S1). To identify differences among rice varieties, the significance probability associated with *F* statistic, labeled “Pr > F,” was calculated by one‐way ANOVA procedure of SAS package. To identify the difference of components between two locations and between 2 years, *p*‐value labeled “Pr>|t|,” which is calculated by paired *t* test procedure using SAS package, was used to examine each environmental factor separately (Supporting Information Table S1). Almost all components showed significant differences among varieties except IDF, proline, S, Cu, Zn, and B2. In terms of locations, 34 out of total 49 components showed significant differences. The components with no differences between two locations were crude fiber, alanine, arginine, aspartam, glycine, histidine, isoleucine, leucine, serine, tyrosine, P, S, miristate, palmitate, and eicosenoate. On the other hand, 27 of total 49 components showed significant differences between two cultivation years. The 99% tolerance intervals with 95% confidence were suggested for each component from the data set to have confidence of true mean (Supporting Information Table S1).

### 3.2 % variability of rice grains nutrients

Natural variation of nutrition composition is the variability occurring naturally because of differences in the genotypes of plants or environmental conditions. For this reason, this study investigated how the composition varies by different environmental effects through several methods of statistical approach. First, the environmental effect on the natural variation of composition was examined by using R statistical procedure. As an appropriate for compositional data from experimental field studies, the linear mixed model in R statistics was used to describe the random effect on the nutritional variation and to state the overall differences and equivalence. The statistical analysis of the experimental data for the compositional assessment is limited to the mean difference and the substantial equivalence over various location and various cultivars. Therefore, a linear mixed model was used for the statistical analysis of compositional data to solve the confidence limits. By using this model, the random effect of environmental factors such as varieties, locations, and cultivation years could be assumed. The variability between variety, location, and year was estimated as related to natural variation in nutrient compositions. For the purpose of modeling the natural variation or variability of components from commercial rice varieties, the individual factors of variety, location, and year, and the mixed variables to interpret the co‐interactions, were subjected to R statistical analysis.

**Figure 1:**
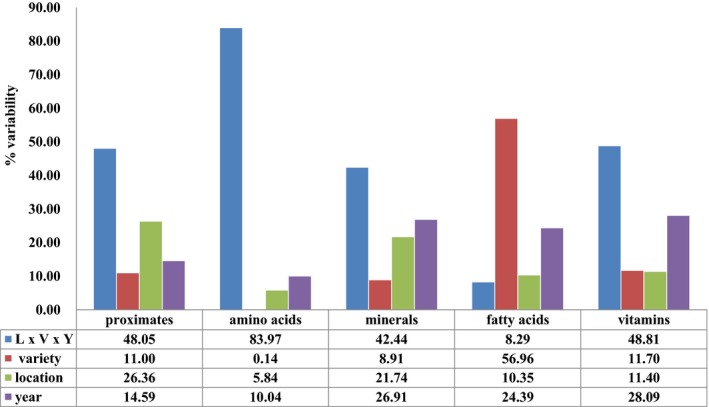
Mean value of the % variability of rice nutrients affected by environmental factors (i.e., variety, location, and year)

The result of % variability for proximate from R statistics according to the environmental effects showed that the natural variation of proximate was mainly contributed by the location (26.36%), followed by the year (14.59%) and variety (11.00%) (Figure 1). Among proximate, moisture (40.92%), protein (67.12%), and carbohydrates (54.83%) were most affected by location effects (Supporting Information Table S2). Crude fat was most affected by the variety (13.69%) while crude fiber and IDF were most affected by year (Supporting Information Table S2). In the results of % variability for amino acids, the year variable most affected the variation of amino acid composition (10.04%), followed by the variables location (5.84%) and variety (0.14%) (Figure 1). Most amino acids were highly affected by the year, especially cysteine, proline, and tyrosine (Supporting Information Table S2). The amino acids most affected by the location were methionine (43.85%) and tryptophan (23.44%) (Supporting Information Table S2). The result of % variability for minerals showed that minerals were most affected by year (26.91%) and next by the location (21.74%) and variety (8.91%). Specifically, the composition variation of S, Cu, and Zn was most highly affected by the year. The minerals affected by the variety rather than other variables were Mg, P, and Mn, while the Ca and Na contents were most affected by the location as compared to other factors. In the result of % variability of fatty acids, the natural variation of fatty acids was mainly contributed by the variety (56.96%), and then by the year (24.39%) and location (10.35%). Most fatty acids were affected by the variety factor, except for the stearate and linolenic acid which were affected by the year factor. In the % variability of vitamins, the natural variation was mainly contributed by the year (28.09%), next by the variety (11.70%), and location (11.40%) (Figure 1). Vit B1 was most highly affected by the variety (44.1%) while B7 was affected by the location (25.90%) (Supporting Information Table S2). By including the interaction of varieties, location, and year in mixed model, the random effect was also assessed to establish equivalence limiting for the average differences over sites, cultivation year, or cultivation year. As a result, mixed variables of location × variety × year (L×V×Y) contributed to the variation of compositions more than the individual variables (Figure 1). The variation for amino acids was highest with 83.97% by L × V × Y, and the variation for other components affected by the L × V × Y was as follows: vitamins (48.81%), proximates (48.05%), minerals (42.44%), and fatty acids (8.29%).

### 3.3 VIP plot by PLS‐DA

**Figure 2:**
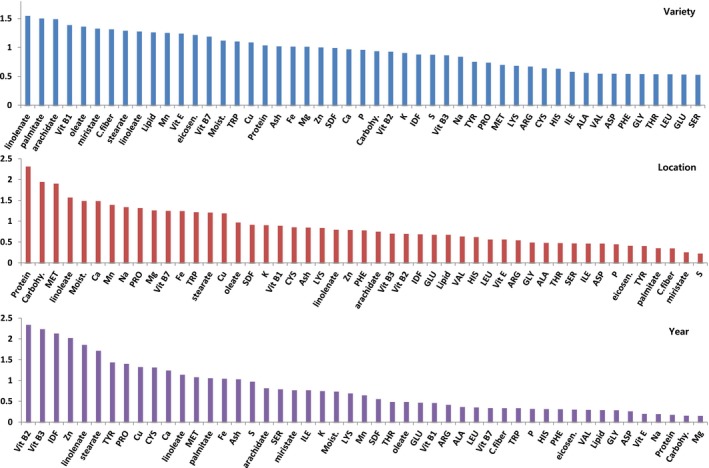
Variable importance in projection (VIP) plot identified by PLS‐DA in a descending order of important nutrients to the variance due to environmental factors (i.e., variety, location, and year)

To identify the compositions which are important to the environmental factors, the VIP plot which was featured by PLS‐DA in a descending order of importance was examined (Figure 2). VIP score refers to the weighted sum of squares of the PLS loadings (Mireia, Stefan, Stefan, & Romà, 2015). VIP analysis was conducted according to environmental factors such as variety, location, and year. As the value of VIP score of components is higher, it can be more important to the applied model. Taking VIP cutoff of around 1.0, the following nutrient compositions were found to be significant among 49 metabolites in the variety model: linolinate, palmitate, arachidate, Vit B1, oleate, miristate, crude fiber, stearate, linoleate, lipid, Mn, Vit E, eicosenoate, Vit B7, moisture, tryptophan, Cu, protein, ash, Fe, Mg, and Zn. Only two features had a VIP score higher than 1.5 that suggested the highest importance of these variables to the variation in the variety model. In the VIP score for the location model, protein, carbohydrates, methionine, linoleate, moisture, Ca, Mn, Na, proline, Mg, Vit B7, Fe, tryptophan, stearate, and Cu had over 1.0 VIP score. Only protein had the VIP score higher than 1.5; therefore, this was most important to the variation in the location model. Under the year model, Vit B2, Vit B3, IDF, Zn, linolenate, stearate, TYR, PRO, Cu, CYS, Ca, linoleate, MET, palmitate, Fe, and Ash had scores over 1.0 VIP. Among these components, Vit B2, Vit B3, IDF, and Zn had VIP scores higher than 1.5, indicating that these were significant to the variation in the year model. Because the VIP analysis result was similar to the % variability from R analysis, it could be concluded that the nutrient compositions most affected by variety were fatty acids, followed by vitamins, proximates, minerals, and amino acids. The nutrient compositions most affected by location were proximates, minerals, vitamins, fatty acids, and amino acids in order while those most affected by year were vitamins, minerals, fatty acids, proximates, and amino acids in order.

### 3.4 PCA results of each compositional nutrient

**Figure 3:**
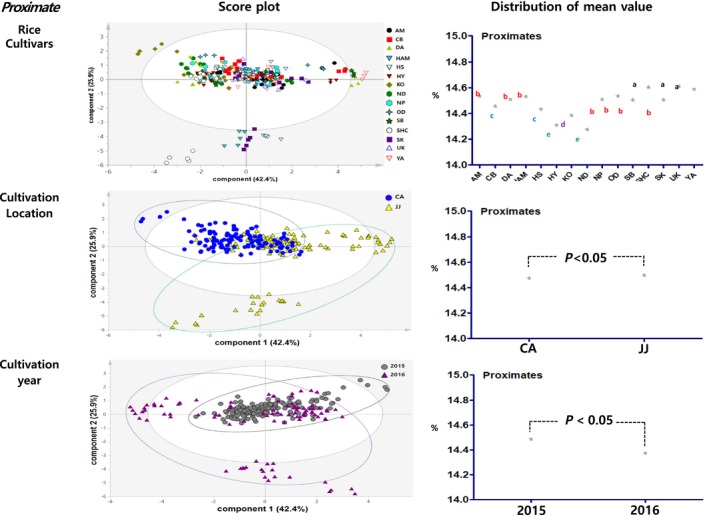
PCA results and mean value distribution for proximate components. PCA, principal component analysis

**Figure 4:**
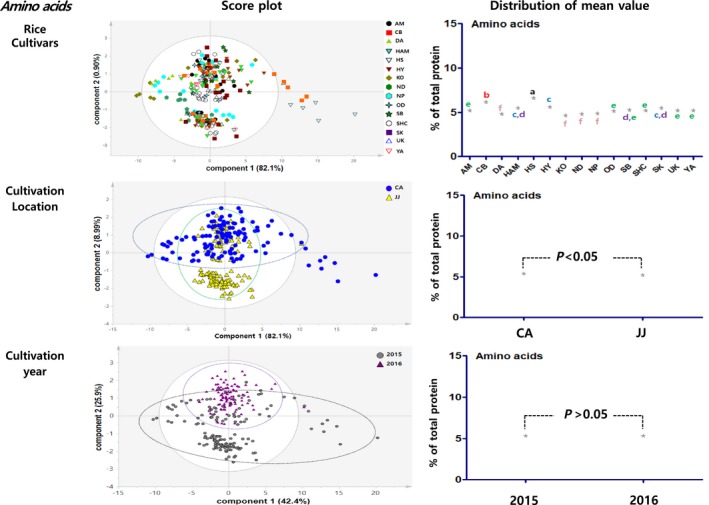
PCA results and mean value distribution for amino acid components

**Figure 5:**
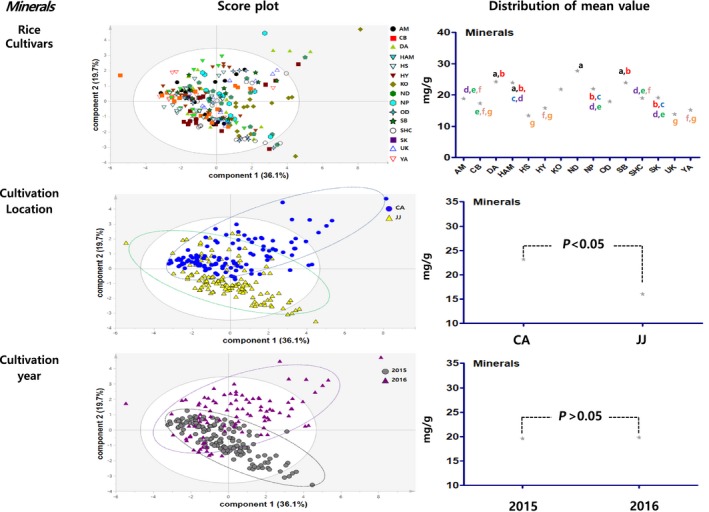
PCA results and mean value distribution for mineral components. PCA, principal component analysis

**Figure 6:**
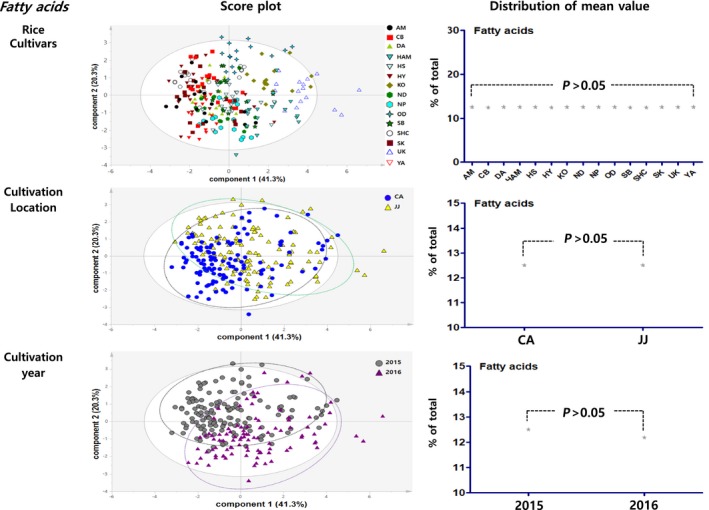
PCA results and mean value distribution for fatty acid components. PCA, principal component analysis

**Figure 7:**
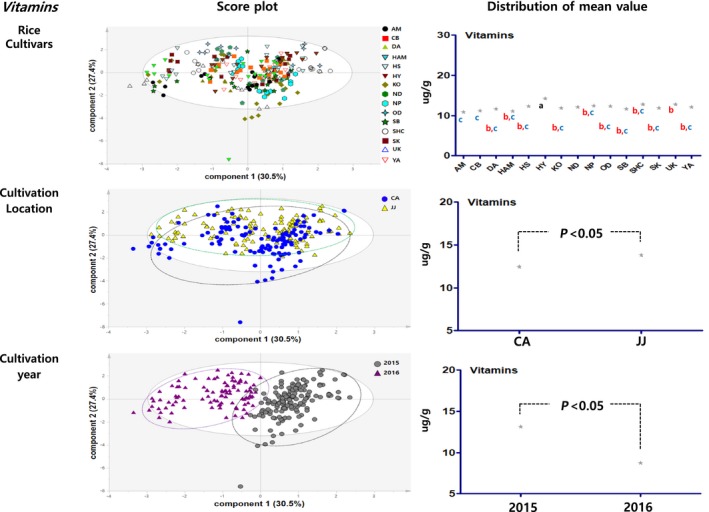
PCA results and mean value distribution for vitamin component. PCA, principal component analysis

Principal component analysis results showed the distribution figure of mean value for the proximate components, amino acids, minerals, fatty acids and vitamins, and were compared by investigating the degree to which they are separated by the factors of cultivars, locations, and cultivation year (Figures 3, 4, 5, 6, 7). The two score vectors were calculated and plotted against each other. The score plot in combination with the loading plot indicated the responsible variables for deviations from normal operation. The mean value distribution figures were used to determine whether there are statistical differences between each variable for visible representation of the degree of separation between the varieties, location, and year variables in the PCA results. The PCA analysis results of proximate components showed that although there was no apparent separation among rice varieties, they were however distinctly separated by the variables of locations and year (Figure 3). From these, it was expected that proximate components could be affected by location and cultivation more than plant varieties. In the distribution of mean value by statistical analysis, proximate component categorized by environmental factors showed significant differences between rice varieties, two locations, and two grown year factors. Although PCA results of amino acids, minerals, and fatty acids did not show any distinct separation by their variety factors, they appeared to be separated by location and year factors (Figures 4, 5, 6). The results of the vitamin PCA analysis showed a distinct separation only by the year factor (Figure 7). In the statistical analysis, the distribution of mean values of amino acids only showed significant differences among their cultivars (Figure 4) while minerals showed significant differences between their cultivars and locations (Figure 5). For the fatty acids, there were no significant differences in all variables (Figure 6). On the other hand, PCA results for vitamins showed no apparent separation by rice cultivars and locations, but the statistical significance test found significant differences between cultivars and location (Figure 7).

## 4 CONCLUSION

### 4.1 Profiles for nutrition composition of 15 rice varieties

The mean values of all nutritional components analyzed in this study were contained within the range of brown rice contents as food presented in the OECD consensus documents (OECD 2004). The nutritional contents of plant foods may vary according to variety and growing conditions. In this study, since rice crops were grown in the same soil and in the same location each year, it was found out that most nutrients were significantly different between varieties more than between locations or between years. In the proximate and mineral results, it was found that the number of significant difference was higher between varieties and between locations than between years. The same result applied for amino acids and fatty acids, which there were more significantly difference between varieties than between year and between location (Table 2). For minerals, the number of nutrients with significant differences showed similarities in the varieties, locations, and years variables. Under the same cultivation conditions, it was found out that the nutritional contents of rice were more varied in different varieties more than in environmental conditions such as cultivation location and year.

**Table 2:** Summary of statistical results for each environmental factor affecting nutritional component: an order of major environmental factors that had an effect on each component

	Counts of significant difference (*p* < 0.05)	Value of % variability	VIP value
Proximate	V = L > Y	L > Y > V	L > V > Y
Amino acids	V > Y = L	Y > L > V	L > Y > V
Minerals	V = L > Y	Y > L > V	L > V > Y
Fatty acids	V > Y > L	V > Y > L	V > Y > L
Vitamins	L > V > Y	Y > V > L	V > Y > L

*Note*

V: variety; L: location; Y: year.

### 4.2 Environmental effects on the natural variation of nutrient components

% variability by R statistics and VIP analysis were performed to determine whether the nutritional content of rice is affected by environmental factors. The statistical analysis of experimental data for the compositional assessment is limited to the mean difference and the substantial equivalence over various location and various cultivars. Therefore, a linear mixed model is used for the statistical analysis of compositional data to solve the confidence limits. The random effect of environmental factors such as varieties, locations, and cultivation years was estimated as a trigger for natural variation in nutrient components. For the purpose of modeling the natural variation or % variability of components from the analyzed data, the individual factor of variety, location, and year and mixed variable of three factors which could be interpreted for the interactions with component variations were introduced into the R statistics. The nutritional component showing the highest natural variation by rice variety factor was fatty acids, followed by vitamins, proximate, minerals, and amino acids. The natural variation of proximate component was mainly contributed to the location factor. In the case of amino acid, minerals, and vitamins, the natural variations were mainly affected by the year factor (Table 2). Each nutrient showed a natural variation caused by environmental factors, and it is believed that various statistical applications can be useful to assess the nutritional safety or equivalence of new plants developed by biological techniques.

### 4.3 Comparison of PCA analyzed according to environmental factors

A PCA analysis was conducted to see how the analyzed component values are separated according to environmental factors such as kind of cultivar, cultivation location, and cultivation year. From the PCA results, the PCA analysis only showed visually that the nutrient components are distinguished by environmental factors. But further statistical analysis is required to conclude whether they differ or not. In this study, ANOVA analysis was applied to complement the PCA results and determine whether they differ or not according to environmental factors.

In conclusion, the natural variations of nutrients caused by the environmental factors were compared by using the % variability, VIP, and PCA analyses. The PCA results could be interpreted as differences between factors (such as between regions, species, and genetic types) together with the result of variance of SAS. The result of % variability with VIP results could be interpreted for natural variations of a component by suggesting the main contributors to nutrient variability. Through these multiple statistical tools, it was found that the natural variation of the nutrients varied depending on the environmental factors.
